# Cu/Zn superoxide dismutase homologs participate in *Nicotiana benthamiana* antiviral responses

**DOI:** 10.3389/fmicb.2025.1561731

**Published:** 2025-07-18

**Authors:** Haijuan Wang, Jidan Zhang, Zhuo Meng, Zhenqi Sun, Dongyang Liu, Bin Li, Fangfang Yan, Chongyi Jia, Hongyou Zhou, Mingmin Zhao

**Affiliations:** ^1^College of Horticulture and Plant Protection, Inner Mongolia Agricultural University, Hohhot, China; ^2^Sichuan Province Company of Tobacco Corporation in China, Chengdu, China; ^3^Agriculture and Animal Husbandry Bureau of Bayannaoer City, Inner Mongolia (Rural Vitalization Bureau), Bayannaoer, China; ^4^Key Laboratory of the Development and Resource Utilization of Biological Pesticide in Inner Mongolia, Hohhot, China

**Keywords:** superoxide dismutases, *NbCu/Zn-SOD*-1, *Nicotiana benthamiana*, tobacco vein mottling virus, antiviral resistance

## Abstract

Superoxide dismutases (SODs) serve as the first line of defense against reactive oxygen species. Copper-zinc superoxide dismutase (*Cu/Zn-SOD*) is an enzyme whose activity depends on copper availability. *Cu/Zn-SOD-1* induction is shown to be involved in the antioxidative and antiviral activity of acetylsalicylic acid in hepatitis C virus (HCV)-expressing cells. Here, RNA sequencing (RNA-seq) analysis identified SOD homologs (three *NbCu/Zn-SOD*, four *NbFe-SOD*, and two *NbMn-SOD*) that were differentially expressed in *Nicotiana benthamiana* during tobacco vein mottling virus (TVMV) infection. *NbCu/Zn-SOD-1* was cloned from *N. benthamiana* and subsequently characterized. Encoding sequence and structural analyses of the *NbCu/Zn-SOD-1* protein confirmed a conserved SOD enzyme domain, GFHLHEfGDtT, indicating that it is SOD-dependent and phylogenetically related to *Cu/Zn-SOD* in *Nicotiana tabacum* (XP 016486719.1). *NbCu/Zn-SOD-1* was primarily localized in the cytoplasm. The transient expression of *NbCu/Zn-SOD-1* led to a reduced accumulation of TVMV and PVY-Rosea1 (PVY-Ros1). In summary, our results suggest that *NbCu/Zn-SOD-1* homologs participate in plant antiviral responses.

## 1 Introduction

Superoxide dismutase (SOD) was discovered in bovine erythrocytes and was officially named by McCord and Fridovich in 1969 (Mccord and Fridovich, 1969). SOD is a key enzyme in the intracellular antioxidant defense system. It catalyzes the dismutation reaction of superoxide anion (O₂^−^) to convert it into hydrogen peroxide (H₂O₂) and oxygen (O₂), which plays a vital role in plant stress resistance (Gupta et al., 2019). Therefore, the balance between intracellular SOD and H_2_O_2_ scavenging enzymes is crucial (Shah et al., 2001). The generated H₂O₂ can be further decomposed into water and oxygen under the influence of other antioxidant enzymes such as catalase (CAT), thereby reducing the intracellular reactive oxygen species (ROS) content, maintaining the intracellular redox balance, and protecting cells from oxidative damage (Yasmeen et al., 2013).

Depending on the metal prosthetic groups that bind to SODs, they are divided into three main types: copper/zinc superoxide dismutase (*Cu/Zn-SOD*), manganese superoxide dismutase (Mn-SOD), iron superoxide dismutase (Fe-SOD) (Zelko et al., 2002). In addition to these three SODs, Ni-SOD has been found in *Streptomyces* and *Streptomyces coelicolor* (Kim et al., 1996). Among them, *Cu/Zn-SOD* is the most abundant SOD in plants and typically plays a role in the cytoplasm, chloroplasts, and extracellular space of eukaryotes. Eukaryotic *Cu/Zn-SOD* is a 32 kDa protein that binds copper and zinc on each subunit. Non-covalent bonds form between subunits, and disulfide bonds and metal ions help stabilize the structure. The amino acid composition of *Cu/Zn-SOD* from different sources was not significantly different, showing clear evolutionary conservation and high sequence homology (Spagnolo et al., 2004). Among these, the sequence identity of SOD in human and bovine erythrocytes is as high as 80%. The highly conserved amino acid sequence of *Cu/Zn-SOD* suggests that it is crucial for maintaining the normal function of the enzyme.

*Cu/Zn-SOD* genes have been isolated from various plants, including longans, bananas, tomatoes, wheat, and *Gossypium hirsutum* (Lin and Lai, 2013; Feng et al., 2015; Feng et al., 2016; Tyagi et al., 2017; Wang et al., 2017). Several studies have demonstrated that *Cu/Zn-SOD* plays a crucial role in plant growth and development, fruit maturation, senescence, and responses to environmental stress, among other processes. In potato plants transfected with the *Cu/Zn-SOD* gene and exposed to salt stress, the activity of the SOD enzyme was significantly higher than in non-transgenic plants, indicating a strong antioxidant capacity and potential for salt tolerance.

In plant viruses, SODs are participated in the infection process. The early accumulation of ROS limits potato virus X (PVX) replication during symptomless extreme resistance (Hernández et al., 2016). A possible biochemical mechanism governing symptomless extreme virus resistance is the accumulation of ROS, which has a dual role in infected plants. Higher concentrations of ROS may promote programmed cell death of infected plant cells as well as the death or limitation of invading pathogens, such as viruses, because of their high toxicity (Halliwell and Gutteridge, 2015). By contrast, at low concentrations, ROS are crucial signaling compounds during the activation of host defense responses, including the induction of antioxidants, in healthy plant cells adjacent to infection sites (Levine et al., 1994; Dat et al., 2000; Thomas et al., 2002; Pogány et al., 2009). Accordingly, it has been known for decades that early (from 6 to 10 h after inoculation) nicotinamide adenine dinucleotide phosphate oxidase-dependent accumulation of superoxide and HO plays a definite role in hypersensitivity-associated resistance to plant viruses such as tobacco mosaic virus (TMV) (Doke and Ohashi, 1988; Rossetti and Bonatti, 2001).

This study revealed differential expression of nine SOD homologs in *Nicotiana benthamiana* during tobacco vein mottling virus (TVMV) infection, specifically identifying three *NbCu/Zn-SODs*, four *NbFe-SODs*, and two *NbMn-SODs*. *NbCu/Zn-SOD-1* was cloned from *N. benthamiana* and further characterized. The effects of *NbCu/Zn-SOD-1* on viral infection with TVMV and PVY-Ros1 were studied.

## 2 Materials and methods

### 2.1 Experimental materials

The vectors pDONR207, pEAQ-HT-DEST3, and *Agrobacterium tumefaciens* C58C1 were provided by Professor Juan Antonio García from the Centre National for Biotechnology (CNB), Spain. *Escherichia coli* DH5α competent cells were purchased from TaKaRa Bio (Osaka, Japan). The infectious clone of TVMV was described previously (Zhao et al., 2020).

### 2.2 Plant preparation

*Nicotiana benthamiana* seeds were sown in soil, and the plants were cultured in a greenhouse at approximately 22–26°C under a 16/8 h light/dark cycle. For infection or mechanical inoculation experiments, *N. benthamiana* plants with the same number of leaves and similar plant sizes (four-to-five leaves) were selected (Pasin et al., 2014).

### 2.3 Identification of SOD homologs from the transcriptome sequencing data

For transcriptome sequencing, *N. benthamiana* plants were infected with TVMV-infected clones, and healthy plants were used as controls (Zhao et al., 2020). Total RNA was extracted using the TRIzol reagent (Thermo Fisher Scientific, Waltham, MA, USA) following the manufacturer’s instructions. Transcriptome sequencing and data analysis were performed. The transcriptome was generated from four complementary DNA (cDNA) libraries, comprising two biological replicates and two treatment sets. Manganese superoxide dismutases (NbSODs) were selected from the transcriptome sequencing data.

### 2.4 *In silico* sequence and structure analysis

A box plot was provided by LC Bio Technology Co., Ltd (Hangzhou, China). The heat map was drawn using TBtools software. Sequences were aligned using ESPript3.x and SnapGene software tools, and evolutionary history was inferred using Molecular Evolutionary Genetics Analysis Version 11 (MEGA 11) software (Kumar et al., 2016). The ExPaSy ProtParam tool was used to analyze the physicochemical properties of *NbCu/Zn-SOD-1*. The secondary structure of *NbCu/Zn-SOD-1* was analyzed using NovoPro. The localization prediction of *NbCu/Zn-SOD-1* using the PlantmPLoc tool and the ScanProsite tool was used to predict the conserved domain of *NbCu/Zn-SOD-1*, and Swiss-Model was used to predict the 3D structural models of remote sensing images (Waterhouse et al., 2018). NP 001234769.2 (*Solanum lycopersicum Cu/Zn-SOD*), XP 015058855.1 (*Solanum pennellii Cu/Zn-SOD*), XP 015164932.1 (*Solanum tuberosum Cu/Zn-SOD*), XP 016547975.1 (*Capsicum annuum Cu/Zn-SOD*), XP 060169665.1 (*Lycium barbarum Cu/Zn-SOD*), CAA32534.1 (*Petunia Cu/Zn-SOD*), *NbCu/Zn-SOD-1* (*N. benthamiana Cu/Zn-SOD*), XP 016486719.1 (*Nicotiana tabacum Cu/Zn-SOD*), BAF 80585.1 (*Populus alba Cu/Zn-SOD*), and NP 001413319.1(*Spinacia oleracea Cu/Zn-SOD*) were used to obtain a structural similarity matrix and gene structure map between *NbCu/Zn-SOD-1* and different species using TBtools software (Chen et al., 2020). A structural dendrogram of *NbCu/Zn-SOD-1* among different species was obtained using MEGA 11 software.

### 2.5 Reverse transcription quantitative polymerase chain reaction (PCR) (RT-qPCR)

Transcript and gene expression levels were measured using fragments per kilobase of transcript per million mapped reads (FPKM). During differential gene expression analysis, the fold change denotes the expression ratio between two groups.

Total RNA was purified from virus-infected leaves and used in cDNA synthesis reactions. For reverse transcription, total RNA (approximately 1 μg) was converted to cDNA using SYBR Green Premix Pro Taq HS qPCR Kit (Rox Plus) (AG11718, Accurate Biology) according to the manufacturer’s instructions. Gene-specific primers (Supplementary Table S1) were designed using QuantStudio™ 3 and 5 Real-Time PCR Systems MAN0010407 (Thermo Fisher Scientific). The expression was normalized using Nbubiquitin as a reference, and the 2−ΔΔCT method was used to calculate the level of gene expression (Yue et al., 2023).

### 2.6 Construction of expression vector of *NbCu/Zn-SOD-1* and its subcellular localization

Gene-specific primers for cloning were designed according to the *NbCu/Zn-SOD-1* sequence (Supplementary Table S1). The *NbCu/Zn-SOD-1* fragment was ligated into the PMD19-T vector and transformed into *E. coli* DH5α competent cells by heat shock, and then plated on Lysogeny Broth (LB) solid medium containing kanamycin and grown at 37°C overnight. Plasmids were extracted using the Tiagen Plasmid Extraction Kit, and restriction enzyme digestion was performed with *Sap* I and *Xba* I. The expression vector pEAQ-*NbCu/Zn-SOD*-1 was constructed using Gateway technology, as previously described (Sun et al., 2024). pDONR-*NbCu/Zn-SOD*-1 was digested with *ApaL* I. pEAQ-*NbCu/Zn-SOD*-1 was digested with *ApaL* I/*Spe*I (Supplementary Figure S1).

The *NbCu/Zn-SOD-1* (663 bp) fragment was amplified from the T-*NbCu/Zn-SOD*-1 plasmid using the 1300GFP-*NbCu/Zn-SOD*-F/R primer. The empty vector, 1,300-GFP-EV, was digested using *BamH* I/*Sal* I to obtain a 10,497 bp fragment. *NbCu/Zn-SOD-1* was ligated into the 1,300-GFP-EV vector to get the expression vector named 1,300-*NbCu/Zn-SOD*-1-GFP. The recombinant plasmids 1,300-*NbCu/Zn-SOD*-1-GFP. The homologous recombination method was used for connection and transformation into *E. coli* DH5a. Identification was performed using *BamH* I/*Spe* I digestion.

### 2.7 Inoculation of the virus

*Agrobacterium* C58C1 cells carrying pEAQ-*NbCu/Zn-SOD*-1, pEAQ-HT-DEST3, or pLX-TVMV were grown to OD_600_ = 1.0. Subsequently, a mixture of pEAQ-*NbCu/Zn-SOD*-1/TVMV was prepared at a V: V ratio of 1:1. As a control, co-infiltration of pEAQ-HT-DEST3 with each virus (V: V = 1:1) was used. Nine days post-inoculation (dpi), symptoms were observed and documented through photography. Three days after the infiltration of *Agrobacterium* carrying pEAQ-*NbCu/Zn-SOD*-1 into *N. benthamiana*, the PVY-Ros1 inoculum preparation in phosphate buffer, and mechanical inoculation were performed as previously described (Pasin et al., 2020).

### 2.8 Western blotting for virus detection

Approximately 0.2 g of infected leaf tissue was frozen in liquid nitrogen, ground into a powder, and added to a solution of 5% SDS, 2 times the volume. The mixture was then mixed thoroughly until homogenization. The mixture was then placed in a water bath at 95°C for 5 min, followed by centrifugation at 12,000 rpm and 4°C for 10 min. The addition of 2 times loading buffer (62.5 mM Tris–HCl, pH 6.8, 25% glycerol, 2% SDS, and 0.01% Bromophenol blue, and 250 mM dithiothreitol) solutions to the serum was performed in a 1:1 ratio. Subsequently, the mixture was reheated in a water bath at 95°C for 5 min and then placed on ice for 2 min. Afterward, it was centrifuged at 12,000 rpm and 4°C for 10 min. The resulting clear upper layer was collected for Western blotting as previously described (Yang et al., 2024).

Immunodetection was conducted using primary antibodies such as anti-TVMV coat protein (CP) for detecting TVMV and anti-PVY CP for detecting PVY-Ros1. Horseradish peroxidase-conjugated goat anti-rabbit immunoglobulin (IgG; ab205718; Abcam) and mouse monoclonal antibodies were used as secondary antibodies. Protein signals were visualized by enhanced chemiluminescence.

### 2.9 Observation of subcellular localization

The infiltrated leaf tissue was cut, and temporary glass slides were made to observe fluorescent protein expression using a laser confocal microscope. The fluorescence of green fluorescent protein (GFP) was visualized with a Leica TCS SP52 confocal laser scanning microscope as described previously (Cheng et al., 2015; Wei and Wang, 2008; Cheng et al., 2015). GFP luminescence was observed at an excitation wavelength of 488 nm.

### 2.10 3,3′-Diaminobenzidine staining

Overexpression of *NbCu/Zn-SOD-1* in *N. benthamiana* plants inoculated with TVMV or PVY-Ros1 was observed. After 9 days, leaves were soaked in 3,3′-diaminobenzidine (DAB) solution (1 mg/mL, pH 3.8), placed under light at 25°C for 12 h, and then transferred to boiling 95% ethanol to remove chlorophyll. After cooling, the leaves were placed in 95% ethanol for 3–5 h.

### 2.11 Determination of SOD enzyme activity

At 1, 3, 6, 9, and 12 days after virus infection, infected leaves were collected to measure the activity of SOD disease-resistance and defense enzymes. The kits (bixbio) were used to measure SOD activity.

## 3 Results

### 3.1 Expression of SOD homologs during TVMV viral infection

**Figure 1:**
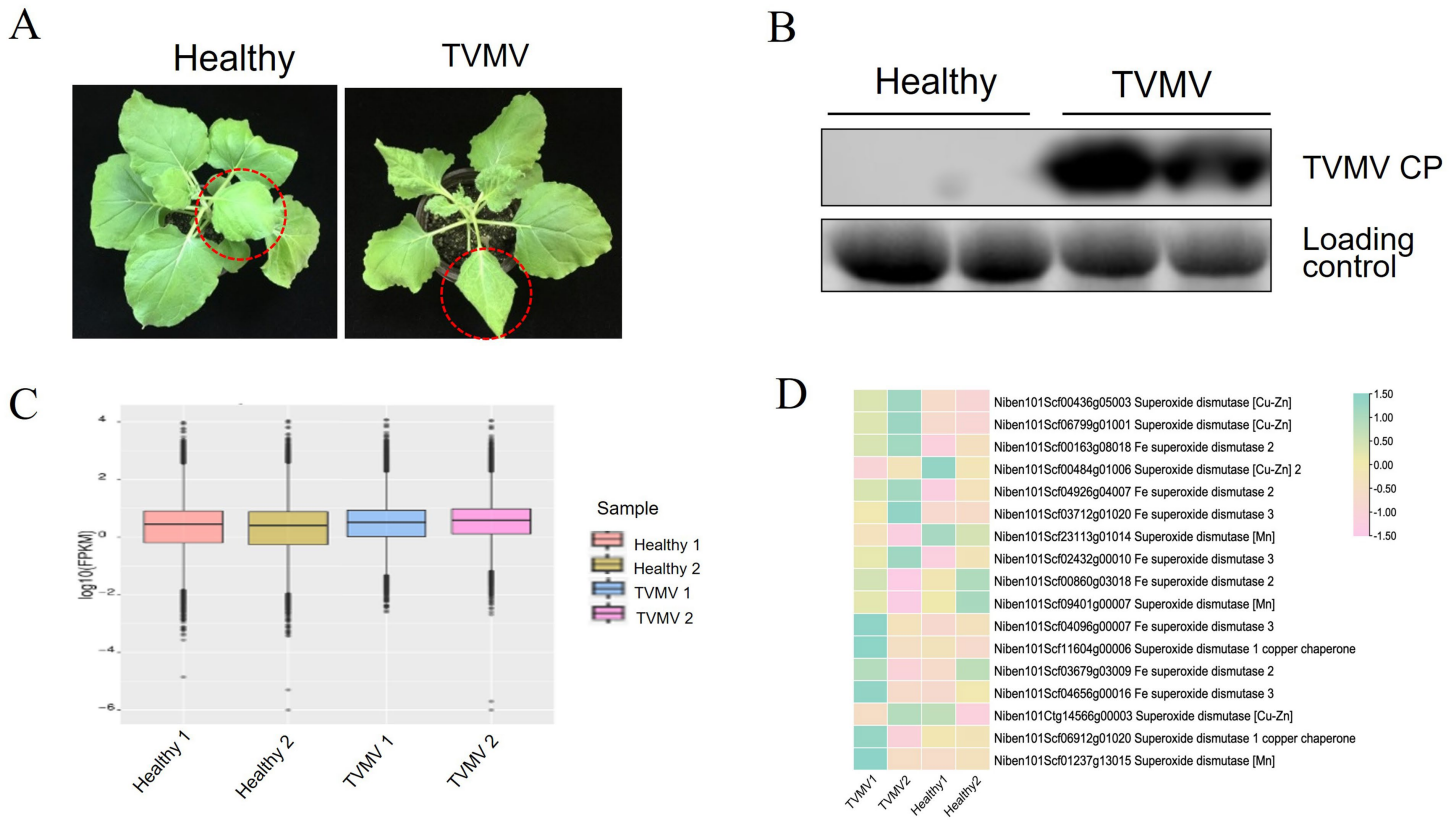
Expression of SOD homologs during TVMV viral infection. **(A)** Phenotypes of TVMV inoculated and control *Nicotiana benthamiana* plants. **(B)** Viral accumulation of TVMV was detected by Western blot with anti-TVMV CP. **(C)** Gene density of cDNA libraries. **(D)** Transcript expression of NbSOD homologs in *Nicotiana benthamiana* analyzed by RNA sequencing (RNA-seq).

The TVMV infectious clone was infiltrated into the leaves of *N. benthamiana* plants with four to five leaves. Symptoms of infection were observed on day 9. Compared with the healthy control, the leaves of plants inoculated with TVMV showed apparent shrinkage and vein mottling (Figure 1A). Viral accumulation was detected by Western blot using a TVMV CP-specific antibody. A high accumulation of TVMV CP was detected in the tested samples, compared with the negative control of healthy plants (Figure 1B). This confirmed the successful infection of TVMV in *N. benthamiana*.

To determine whether SOD homologs were regulated by TVMV infection, we performed a transcriptome sequencing assay using samples from *N. benthamiana* plants infected with TVMV at 9 dpi. The boxplot results showed that the TVMV treatment and healthy control groups had high repeatability (Figure 1C), suggesting the reliability of the data and enabling them to be used for further analysis. We found that 17 transcripts encoding SOD homologs were differentially expressed in TVMV-infected samples compared with healthy control plants (Table 1; Figure 1D).

**Table 1:** Differential expression of SOD homoogues in in *Nicotiana benthamiana* infected with TVMV.

Gene_ID	Description	FPKM. TVMV1	FPKM. TVMV2	FPKM. Healthy1	FPKM. Healthy2	fc	log2 (fc)	pval
Niben101Scf00436g05003	Superoxide dismutase [Cu-Zn]	82.69	117.82	39.47	27.48	3.00	1.58	0.00
Niben101Scf06799g01001	Superoxide dismutase [Cu-Zn]	21.59	30.62	11.73	9.72	2.43	1.28	0.00
Niben101Scf00163g08018	Fe superoxide dismutase 2	11.62	14.93	4.92	7.97	2.06	1.04	0.01
Niben101Scf00484g01006	Superoxide dismutase [Cu-Zn] 2	13.00	18.17	29.69	18.66	0.64	−0.63	0.01
Niben101Scf04926g04007	Fe superoxide dismutase 2	19.97	24.90	9.39	15.43	1.81	0.85	0.03
Niben101Scf03712g01020	Fe superoxide dismutase 3	4.26	6.93	2.79	3.00	1.93	0.95	0.03
Niben101Scf23113g01014	Superoxide dismutase [Mn]	9.49	7.75	12.23	10.98	0.74	−0.43	0.08
Niben101Scf02432g00010	Fe superoxide dismutase 3	14.37	17.83	9.70	13.06	1.41	0.50	0.26
Niben101Scf00860g03018	Fe superoxide dismutase 2	287.40	208.39	263.43	309.29	0.87	−0.21	0.26
Niben101Scf09401g00007	Superoxide dismutase [Mn]	135.31	123.79	134.10	141.72	0.94	−0.09	0.41
Niben101Scf04096g00007	Fe superoxide dismutase 3	13.82	9.87	8.78	9.74	1.28	0.36	0.53
Niben101Scf11604g00006	Superoxide dismutase 1 copper chaperone	30.68	20.57	21.72	19.66	1.24	0.31	0.53
Niben101Scf03679g03009	Fe superoxide dismutase 2	622.52	517.25	536.92	614.76	0.99	−0.01	0.60
Niben101Scf04656g00016	Fe superoxide dismutase 3	6.23	3.71	3.59	4.51	1.23	0.30	0.68
Niben101Ctg14566g00003	Superoxide dismutase [Cu-Zn]	52.83	63.61	62.29	48.40	1.05	0.07	0.81
Niben101Scf06912g01020	Superoxide dismutase 1 copper chaperone	17.53	11.58	14.03	13.71	1.05	0.07	0.87
Niben101Scf01237g13015	Superoxide dismutase [Mn]	57.84	47.03	46.68	48.21	1.11	0.14	0.96

### 3.2 Identification of *NbCu/Zn-SOD-1* in *Nicotiana benthamiana*

**Figure 2:**
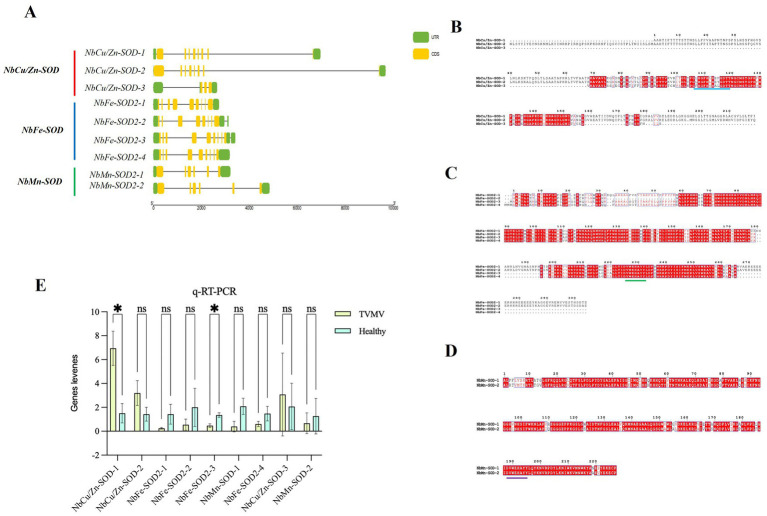
Identification of *NbCu/Zn-SOD* in *Nicotiana benthamiana* infected with TVMV. **(A)** The CDS-UTR structures and the multiple sequence alignment of SOD homologs in *N. benthamiana*. Yellow rectangles represent CDS, black lines represent exons, and green rectangles represent untranslated regions. **(B)** The multiple sequence alignment of *Cu/Zn-SOD* homologs of *N. benthamiana*; Dashes between amino acid alphabets indicate gaps that were introduced to optimize alignment; The background colors of alphabets represent the higher (white) to lower (red) variability of certain sites in sequences; The blue lines represent the shared conserved sequences. **(C)** The multiple sequence alignment of Fe-SOD homologs; The green lines represent the shared conserved sequences. **(D)** The multiple sequence alignment of Mn-SOD homologs; The purple line represents the shared conserved sequence. **(E)** Relative expression of SOD homologs after TVMV infection of *N. benthamiana* by RT-qPCR. *p < 0.05 and “ns” represents *p* > 0.5 by Student’s *t*-test.

Nine SOD homologs, including three *NbCu/Zn-SOD*, four *NbFe-SOD2* and two *NbMn-SOD* homologs, were analyzed using RT-qPCR. The expression of three SODs (*NbCu/Zn-SOD-1*, *NbCu/Zn-SOD-2*, and *NbCu/Zn-SOD-3*) was upregulated and that of six SODs (*NbFe-SOD2-1*, *NbFe-SOD2-2*, *NbFe-SOD2-3*, *NbFe-SOD2-4*, *NbMn-SOD-1*, and *NbMn-SOD-2*) was downregulated after TVMV infection. The expression of *NbCu/Zn-SOD-1* was significantly upregulated, whereas that of *NbFe-SOD2-3* was significantly downregulated (Figure 2A). The amino acid composition of the SOD homologs ranged from 101 to 305 amino acids. The relative molecular weights of SOD homologs ranged from 10.3 34.9 kD. Among them, *NbFe-SOD2-3*, *NbMn-SOD-1*, and *NbMn-SOD-2* were basic proteins with isoelectric points greater than 7, whereas the other six proteins were acidic proteins with isoelectric points below 7. Hydrophilicity analysis revealed that the Grand Average of Hydropathicity (GRAVY) values of the SOD proteins were negative, indicating they were classified as hydrophilic proteins. Prediction of subcellular localization showed that *NbMn-SOD-1* and *NbMn-SOD-2* were localized in the mitochondria, whereas other SOD homologs were localized in the chloroplasts (Table 2).

**Table 2:** The characteristics of SOD homologues in *Nicotiana benthamiana* and predicted subcellular localization.

Gene_ID	Name	Subgroup	Amino acid length	P I	Mw (Da)	GRAVY	Subcellular localization
Niben101Scf00436g05003	*NbCu/Zn-SOD*-1	*Cu/Zn-SOD*	220	6.09	22577.35	−0.087	Chloroplast
Niben101Scf06799g01001	*NbCu/Zn-SOD*-2	*Cu/Zn-SOD*	271	6.54	29061.89	−0.229	Chloroplast
Niben101Scf00163g08018	NbFe-SOD2-1	Fe-SOD	305	5.65	34981.40	−0.607	Chloroplast
Niben101Scf04926g04007	NbFe-SOD2-2	Fe-SOD	278	5.21	31997.70	−0.638	Chloroplast
Niben101Scf00860g03018	NbFe-SOD2-3	Fe-SOD	250	7.10	27937.67	−0.331	Chloroplast
Niben101Scf09401g00007	NbMn-SOD-1	Mn-SOD	228	7.85	25505.13	−0.339	Mitochondrion
Niben101Scf03679g03009	NbFe-SOD2-4	Fe-SOD	249	6.71	28037.80	−0.388	Chloroplast
Niben101Ctg14566g00003	*NbCu/Zn-SOD*-3	*Cu/Zn-SOD*	101	5.24	10318.30	−0.196	Chloroplast
Niben101Scf01237g13015	NbMn-SOD-2	Mn-SOD	228	8.50	25512.22	−0.336	Mitochondrion

The coding sequence (CDS)-untranslated region structure and multiple sequence alignments of SOD homologs in *N. benthamiana* were profiled. The length of the SOD locus in *N. benthamiana* was 2,658 to 9,691 bp. Three *NbCu/Zn-SOD* homologs contained three to nine CDS regions, and four *NbFe-SOD2* homologs contained eight to nine CDS regions. *NbMn-SOD* homologs contained six CDS (Figure 2B). The amino acid sequences of SOD homologs of *N. benthamiana* were aligned using multiple sequence alignments. There was a conserved sequence “GFHLHEfGDtT” and “GFHVHAlGDtT” in the *Cu/Zn-SOD* amino acid sequence of *N. benthamiana* (Figure 2C). A conserved sequence “DvWEHAYY” was distributed on the amino acid sequence of *Fe-SOD* (Figure 2D). A conserved sequence “DvWEHAYY” was distributed in the amino acid sequence of *Mn-SOD*, which was the same as the conserved sequence distributed in the amino acid sequence of *Fe-SOD* (Figure 2E).

### 3.3 Characteristic of *NbCu/Zn-SOD-1* in *Nicotiana benthamiana*

**Figure 3:**
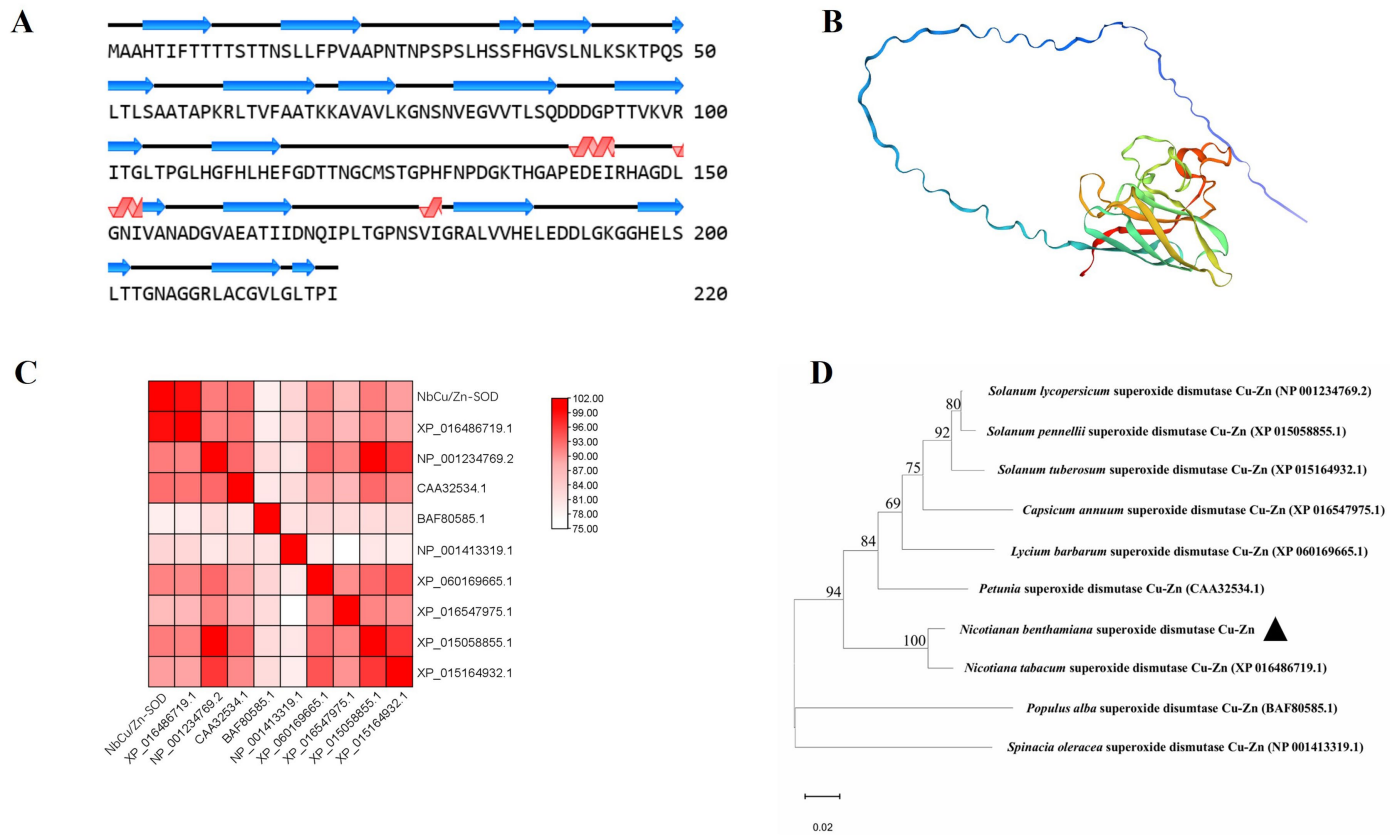
Structural modeling and analysis of *NbCu/Zn-SOD-1*. **(A)** Secondary structures derived from the predicted models of *NbCu/Zn-SOD-1*. **(B)** Tertiary structural models of *NbCu/Zn-SOD-1*; colors by prediction confidence scores. **(C)** The structural similarity matrix of *NbCu/Zn-SOD-1*; colors by Dali Z-scores. **(D)** The amino acid structural dendrogram of *NbCu/Zn-SOD-1*; branch support values are shown. Structural models of *NbCu/Zn-SOD-1* were computed by MEGA 11.

The secondary structure of *NbCu/Zn-SOD-1* was analyzed using NovoPro. Sixteen *α*-helix and three *β*-folded structures were identified from the structure of *NbCu/Zn-SOD-1*(Figure 3A). The three-dimensional structure of *NbCu/Zn-SOD-1* predicted by Swiss-Model showed a conserved core with high prediction confidence scores, which was flanked by less conserved termini with a variable number of disordered residues (Figure 3B). To confirm the structural correlation between the cloned *NbCu/Zn-SOD-1* homologs and known *Cu/Zn-SOD* genes from other plant species, a reference structure obtained by X-ray crystallography or high-resolution modeling was used for structural comparison and phylogenetic tree alignment analysis. The structural similarity matrix and evolutionary tree showed that *NbCu/Zn-SOD-1* was highly similar to the *Cu/Zn-SOD* of *Nicotiana tabacum* (Figures 3C,D).

### 3.4 Subcellular localization of *NbCu/Zn-SOD-1* in *Nicotiana benthamiana*

**Figure 4:**
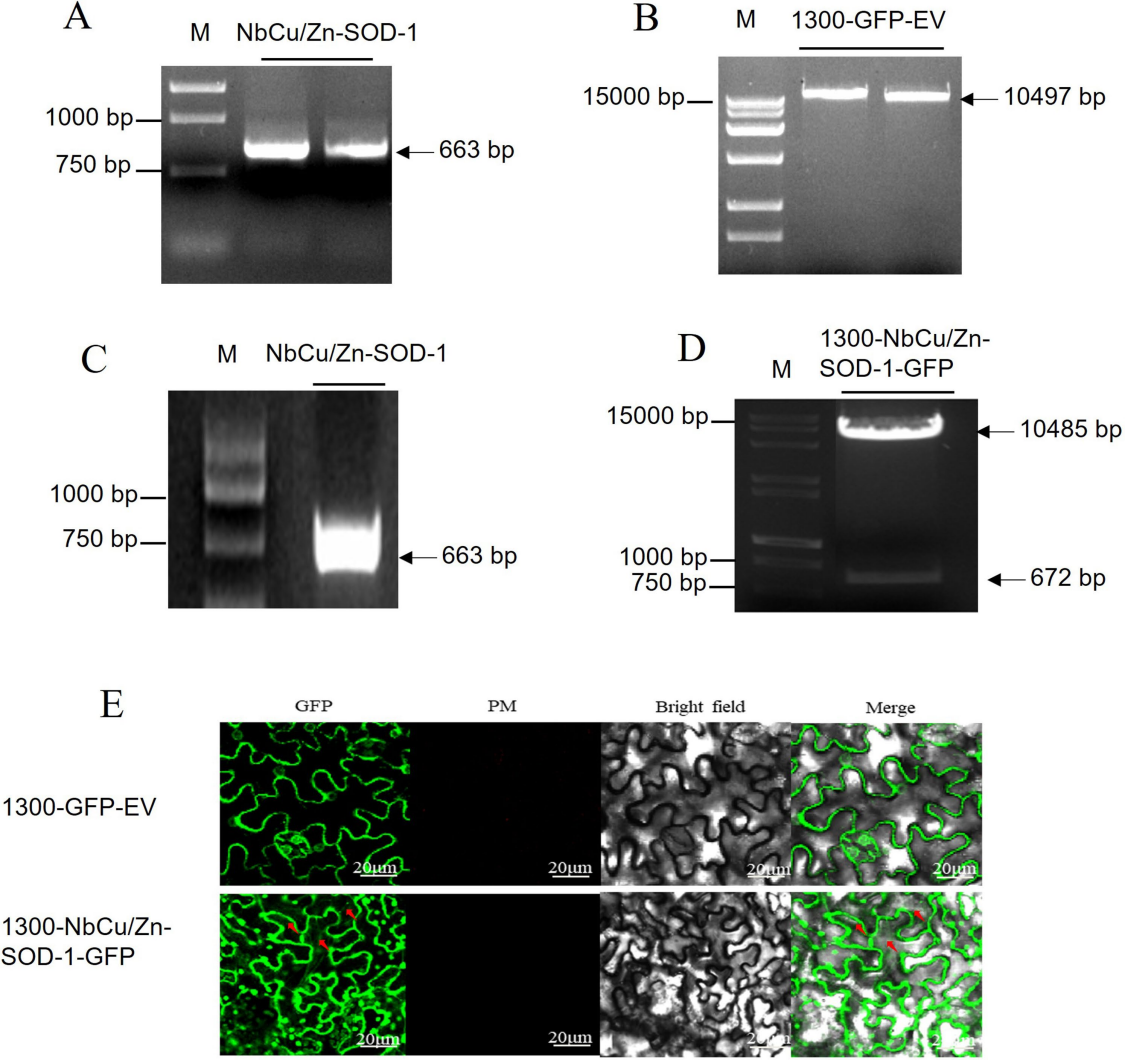
Construction of *NbCu/Zn-SOD-1* subcellular vector and observation with subcellular localization. **(A)** *NbCu/Zn-SOD-1* fragments obtained by PCR with primer 1,300-*NbCu/Zn-SOD*-F/R in agarose gel. **(B)** Digestion of 1,300-GFP-EV by *BamH* I*/Sal* I. **(C)** 1,300-*NbCu/Zn-SOD*-1-GFP fragments were amplified by PCR in *Escherichia coli* DH5a. **(D)** Digestion of 1,300-*NbCu/Zn-SOD*-1-GFP by *BamH* I*/Spe* I. Fragment sizes of the marker are shown on the right. **(E)** GFP luminescence was observed at an excitation wavelength of 488 nm. The 1,300-GFP-EV empty vector was used as a control.

To determine the subcellular localization of *NbCu/Zn-SOD-1* in plant cells, we cloned the *NbCu/Zn-SOD-1* sequence into the recombinant vector 1,300-GFP-EV. The *NbCu/Zn-SOD-1* (663 bp) fragment was amplified from healthy *N. benthamiana* (Figure 4A). The vector 1,300-GFP-EV was digested using *BamH* and I/*Sal* I to obtain a 10,497 bp fragment (Figure 4B). *NbCu/Zn-SOD-1* was ligated into the 1,300-GFP-EV vector to get the expression vector named 1,300-*NbCu/Zn-SOD*-1-GFP. The recombinant plasmid 1,300-*NbCu/Zn-SOD*-1-GFP was transformed into *E. coli* DH5a and verified by PCR (a 663 bp fragment) (Figure 4C) and restriction enzyme digestion with *BamH* I/*Spe* I (10,485 bp and 672 bp) (Figure 4D). The plasmid 1,300-NbCu /Zn-SOD-1-GFP was transformed into *Agrobacterium* C58C1 cells and infiltrated into *N. benthamiana*. Fluorescence was observed 3 dpi. GFP fluorescence was detected in the cytoplasm, indicating that *NbCu/Zn-SOD-1* was localized in the cytoplasm (Figure 4E).

### 3.5 Effect of *NbCu/Zn-SOD-1* on plant viral infection

**Figure 5:**
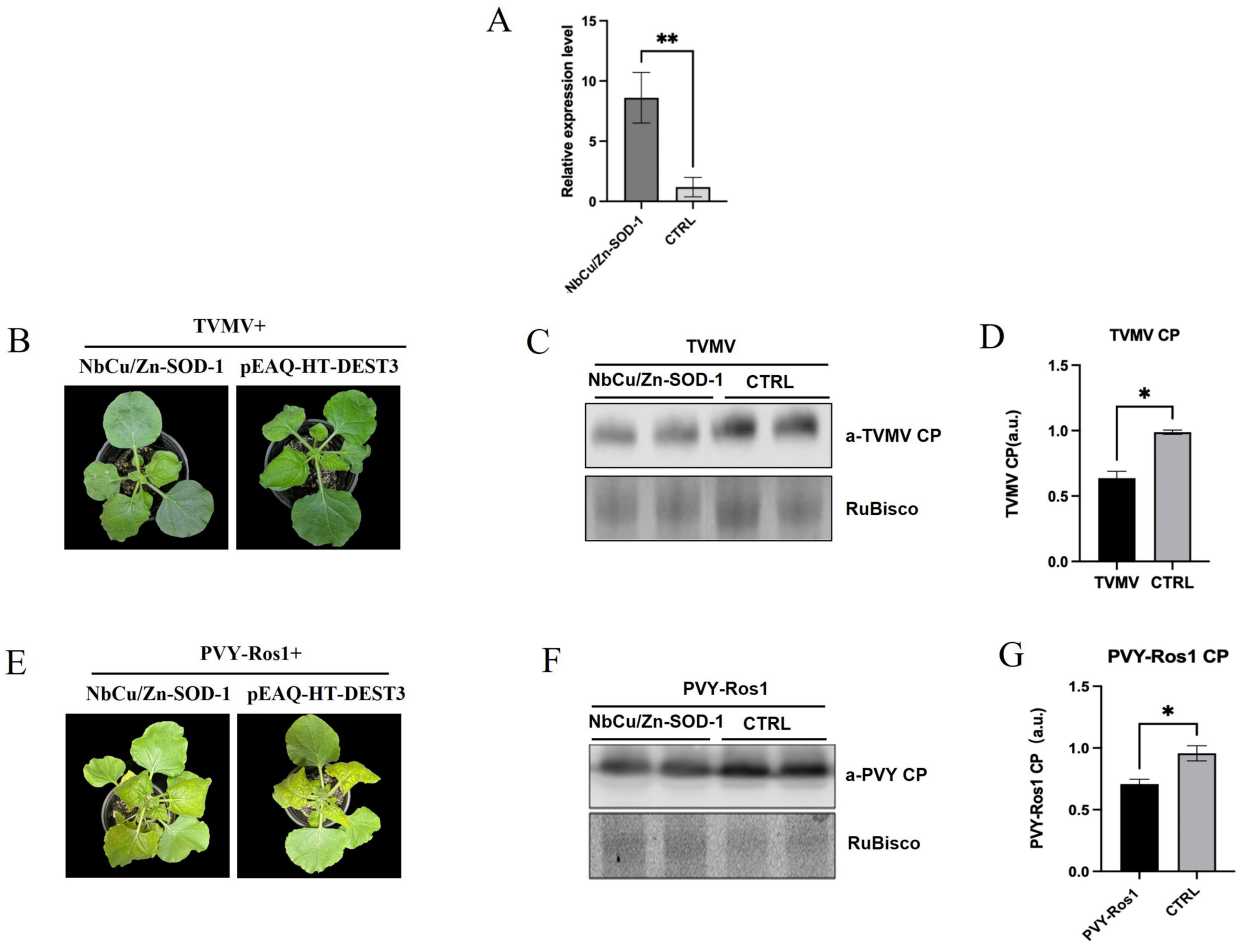
Effect of *NbCu/Zn-SOD-1* on plant viral infection. **(A)** Plots show RT-qPCR transcript quantification values [mean ± standard deviation (SD)] in samples collected from leaves infiltrated with *NbCu/Zn-SOD-1* overexpressing constructs. **p < 0.01 and *p < 0.05 by Student’s *t*-test. **(B)** Total plants and systemic leaves of overexpression *NbCu/Zn-SOD-1* and empty vector (pEAQ-HT-DEST3) plants infected with TVMV as observed at 9 days post-inoculation (dpi). **(C)** Viral protein accumulation in upper uninoculated systemic leaves was measured by Western blot with an anti-TVMV coat protein (CP) serum; RuBisCO large subunit is shown as a loading control. **(D,G)** Plots show signal quantification values (mean ± SD). CTRL, control condition; *p* values by Student’s *t*-test are shown. **(E)** Total plants and systemic leaves of overexpression *NbCu/Zn-SOD-1* and empty vector (pEAQ-HT-DEST3) plants infected with PVY-Ros1 as observed at 9 days post-inoculation (dpi). **(F)** Western blot with an anti-PVY coat protein (CP) serum.

To determine the effect of *NbCu/Zn-SOD-1* on plant viral infection, the *NbCu/Zn-SOD-1* (663 bp) fragment from *N. benthamiana* was amplified and verified by sequencing (Supplementary Figures S1 and S2). The fragment of *NbCu/Zn-SOD-1* was cloned into pEAQ-HT-D3 using a gateway recombination system to obtain pEAQ-*NbCu/Zn-SOD*-1. The plasmid pEAQ-*NbCu/Zn-SOD*-1 was transformed into *Agrobacterium* C58C1 and infiltrated into *N. benthamiana* leaves. The expression levels of *NbCu/Zn-SOD-1* were detected using RT-qPCR. We found that the expression of *NbCu/Zn-SOD-1* was significantly increased compared with that in healthy controls (Figure 5A). Thus, the transient expression of *NbCu/Zn-SOD-1* was functional.

pEAQ-*NbCu/Zn-SOD*-1 was then co-infected with the TVMV infectious clone. The upper leaves of TVMV-infected *N. benthamiana* displayed slight symptoms of shrinkage, while the control plants exhibited significantly stronger symptoms (Figure 5B). Virus accumulation in the upper leaves was analyzed by Western blotting 9 dpi. Western blot analysis showed that TVMV accumulation was significantly reduced in *NbCu/Zn-SOD-1* overexpressing plants compared with the controls (Figures 5C,D). Three days after pEAQ-*NbCu/Zn-SOD*-1 overexpression, PVY-Ros1 was inoculated by mechanical inoculation, and symptoms of the plants were observed after 9 days. The number of plants overexpressing *NbCu/Zn-SOD-1* was significantly reduced compared with the control (Figure 5E). Western blotting results were similar to those of TVMV, and the accumulation of PVY-Ros1 was reduced in plants overexpressing *NbCu/Zn-SOD-1* compared with the control (Figures 5F,G). This suggests that *NbCu/Zn-SOD-1* has a pronounced inhibitory effect on viral infection.

### 3.6 *NbCu/Zn-SOD-1* enhances H_2_O_2_ quenching by increasing superoxide dismutase activity

**Figure 6:**
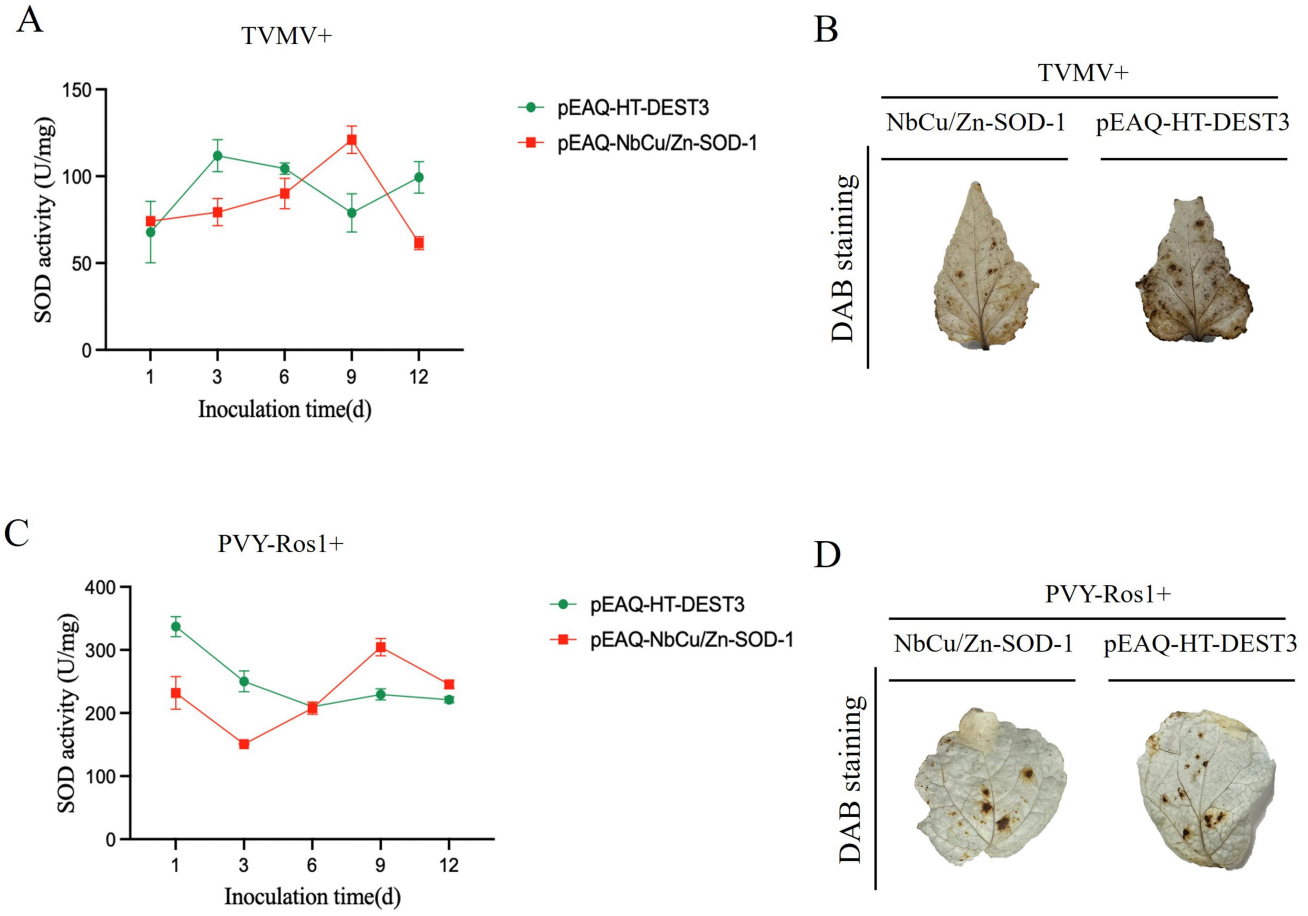
Effects of transient expression of *NbCu/Zn-SOD-1* gene on SOD enzyme activity and ROS after TVMV and PVY-Ros1 infection. **(A–C)** Changes of SOD activity in *Nicotiana benthamiana* leaves with transient *NbCu/Zn-SOD-1* gene after TVM or PVY-Ros1 inoculation. **(B–D)** After overexpression of *NbCu/Zn-SOD-1*, TVMV or PVY-Ros1 was inoculated, and the leaves of the system were taken to detect ROS in *N. benthamiana* leaves *in situ*. Leaves of a tobacco system inoculated with TVMV with an empty vector were used as controls.

After transient expression of *NbCu/Zn-SOD-1* gene, SOD resistance defense enzyme activities in the leaves were detected after TVMV or PVY-Ros1 inoculation. The results showed that after co-injection of *NbCu/Zn-SOD-1* gene and TVMV, SOD activity in plants increased from 1 to 9 days, reaching the highest value of 128.9 U/g on day 9, after which enzyme activity began to decline (Figure 6A). ROS accumulation in TVMV-infected plants was monitored using DAB staining, which showed lower levels of H_2_O_2_ in TVMV-infected plants than in control plants (Figure 6B). Three days after overexpressing *NbCu/Zn-SOD-1* gene, *N. benthamiana* plants were mechanically inoculated with PVY-Ros1, and the upper leaves were used to detect SOD enzyme activity. SOD activity in plants reached the highest value of 318.2 U/mg on day 9, after which enzyme activity decreased slightly (Figure 6C). ROS accumulation in PVY-Ros1-infected plants was monitored using DAB staining, and the results showed no significant difference in H_2_O_2_ levels between PVY-Ros1-infected plants and control plants (Figure 6D). Transient expression of *NbCu/Zn-SOD-1* gene increased SOD activity in *N. benthamiana*, thereby improving the defense of *N. benthamiana* against virus infection.

## 4 Discussion

ROS are produced during the plant life processes. Superoxide anions form H_2_O_2_ under the catalysis of SOD, and H_2_O_2_ forms O_2_ through the catalysis of CAT (Fu et al., 2025). Under normal conditions, ROS in the plant are in a dynamic and stable state of production and clearance (Chen et al., 2022). When the plant is under stress, this balance is disrupted, and numerous ROS are produced, resulting in damage to the plant. Transgenic tobacco plants showed higher water content, less electron damage, lower malondialdehyde content, higher antioxidant enzyme activity, and higher survival rates than non-transgenic plants after prolonged drought and salt stress (Negi et al., 2015). When plants are infected with viruses, the redox reaction balance in the cells is disrupted, and many reactive oxygen species, such as O₂^−^ and H₂O₂, are produced. These ROS attack lipids, proteins, and nucleic acids, leading to cell membrane damage, enzyme inactivation, and DNA mutations, which in turn promote virus propagation (Paolo et al., 2016).

ROS-scavenging systems play a crucial role in cellular functions because ROS are highly cytotoxic. The synchronous action of SOD, CAT, ascorbate peroxidase, monodehydroascorbate reductase, dehydroascorbate reductase, and glutathione reductase is part of the antioxidant system that protects plants from ROS damage (Kozi, 1999; Scandalios, 2005; Ashraf, 2009; Gill and Tuteja, 2010). Plants with high expression of these antioxidant enzymes are more tolerant to salt and oxidative stress. It has been shown that salt stress alters the levels of antioxidant enzymes to a large extent, providing tolerance to plants (Kim et al., 2005; Noreen and Ashraf, 2009; Wang et al., 2009; Tarchoune et al., 2010).

*Cu/Zn-SOD* plays a protective role in various types of tissue, protecting them from oxidative damage (Lewandowski et al., 2019). It is a critical enzyme in limiting reactive oxygen species in both the cytosol and the mitochondrial intermembrane space (Fetherolf et al., 2017). It plays a vital role in the balance of oxidation and antioxidants in the body, and is closely related to the occurrence and development of many diseases. Low concentrations of ROS play an essential role in plant disease resistance signaling. *Cu/Zn-SOD* may affect downstream signaling pathways by regulating ROS levels, such as activating mitogen-activated protein kinase cascade, or inducing the expression of genes related to disease resistance (Sumino et al., 2020). *Cu/Zn-SOD* may inhibit viral replication, assembly, or release by affecting the redox reaction state in cells. For example, certain viruses require a specific oxidative environment for replication, and *Cu/ Zn-SOD* mediated antioxidant effects may disrupt this environment, thereby limiting virus proliferation (Teoh et al., 2003).

In this study, we identified genes that were differentially expressed during TVMV infection, from which SOD homologs (three *NbCu/Zn-SOD*, four *NbFe-SOD*, and two *NbMn-SOD*) were selected. The qPCR results showed that the expression of *NbCu/Zn-SOD*-1 was significantly upregulated in tobacco plants infected with TVMV, with notable differences. This suggests that *Cu/Zn-SOD-1* may prevent the harmful effects of ROS accumulation in plants and that SOD may play a key role in the antiviral process in plants.

In this study, the *NbCu/Zn-SOD-1* gene was cloned, and its sequence was analyzed. The presence of the SOD enzyme activity domain was confirmed and was supported by secondary and tertiary structure prediction and modeling. The conserved domains are located in the central portion of these proteins. Phylogenetic analysis revealed that *NbCu/Zn-SOD-1* was clustered with *Cu/Zn-SOD* (XP 016486719.1). This suggests that they may have engaged in similar activities. *NbCu/Zn-SOD-1* subcellular localization results showed that *NbCu/Zn-SOD-1* was localized in the cytoplasm.

We further investigated their roles in plant viral infections. The results showed that transient expression of *NbCu/Zn-SOD-1* resulted in a decrease in the accumulation of TVMV and PVY-Ros1. This suggests that *NbCu/Zn-SOD-1* homologs are involved in plant antiviral responses. Therefore, exploring the potential applications of *NbCu/Zn-SOD-1* through bioengineering is an efficient way to improve anti-viral resistance in plants.

## Data Availability

The data supporting the findings of this study are available in the article and its Supplementary material or from the corresponding authors. The cloned gene sequences are available at NCBI (*NbCu/Zn-SOD-1*).
