# Supplementary material for: Cu/Zn superoxide dismutase homologs participate in Nicotiana benthamiana antiviral responses
Source: Front Microbiol. 2025 Jul 18;16:1561731. doi: 10.3389/fmicb.2025.1561731 (PMC12313585; doi:10.3389/fmicb.2025.1561731)

**Figure S1.** Amplification and expression vector construction of *NbCu/Zn-SOD-1*.

**(A)** *NbCu/Zn-SOD-1* fragments obtained by PCR with primer NbCu/Zn-SOD-F/R in agarose gel. **(B)** *NbCu/Zn-SOD-1* fragments were ligated to the pMD19-T vector, and the fragments were amplified by PCR in *E. coli* DH5a. **(C)** Digestion profiles of purified plasmids from selected *Escherichia coli* colonies. The T-NbCu/Zn-SOD-1 plasmid was used as a control. DNA sizes of marker are shown on the right. The size of *Sap* I */ Xba* I digestion fragments are shown on the right. **(D)** *NbCu/Zn-SOD-1* fragments were ligated to the pDONR207 vector. *ApaL* I digestion of plasmids pDONR-NbCu/Zn-SOD-1. The size of fragments are shown on the right. **(E)** *NbCu/Zn-SOD-1* fragments were ligated to the pEAQ-DEAT3 vector. *ApaL* I */ Spe* I digestion of plasmids pEAQ-NbCu/Zn-SOD-1. The size of fragments are shown on the right.


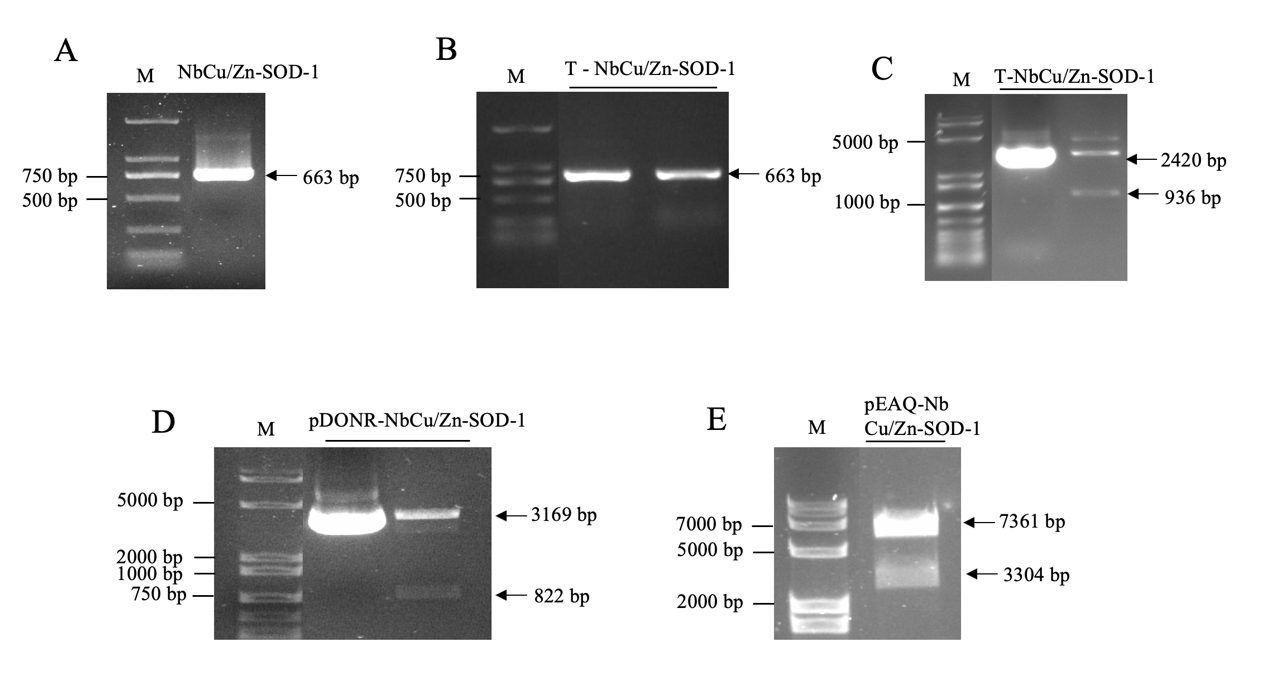


**Figure S2.** Sequence map of *NbCu/Zn-SOD-1*.


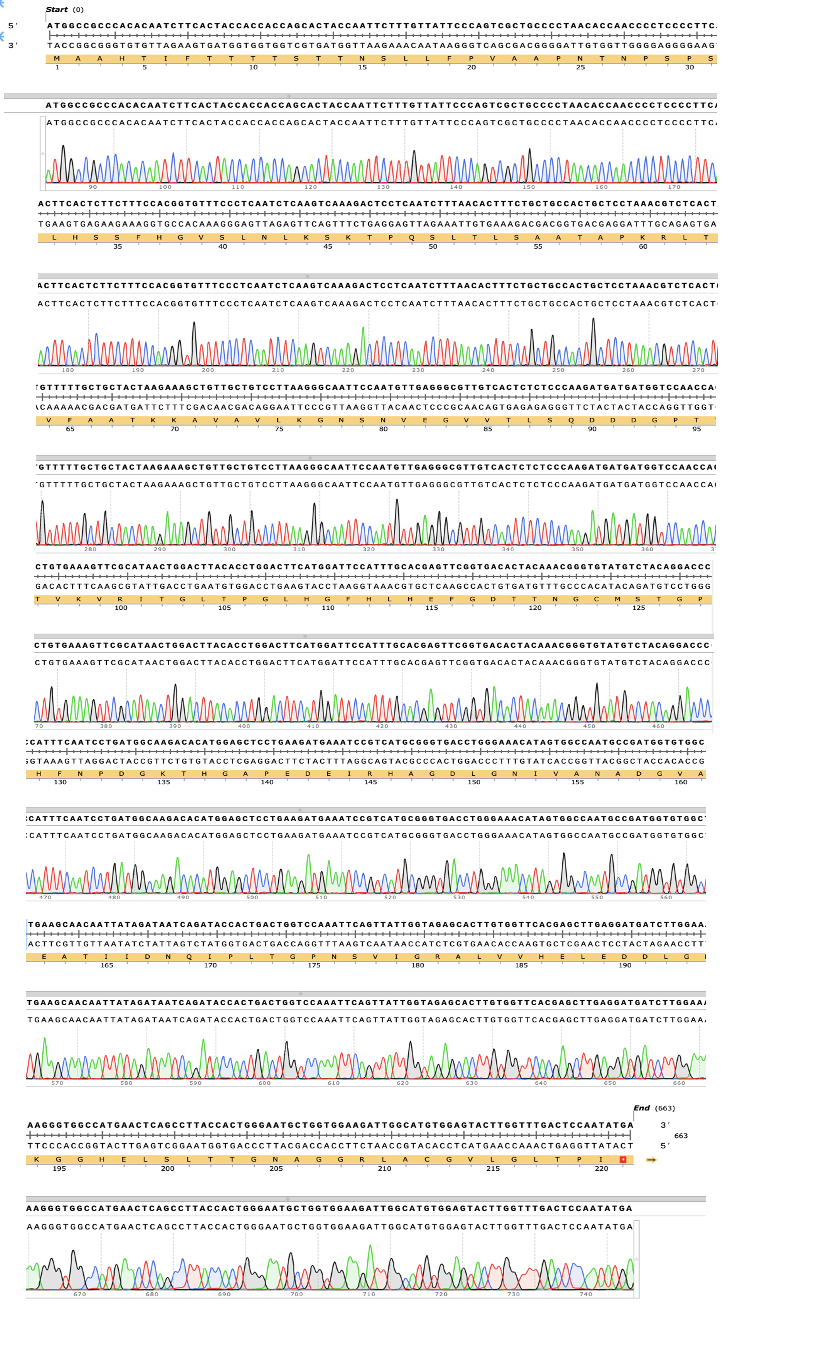

Supplement: Supplementary file 1 [file Supplementary_file_1.docx]
