# Supplementary material for: Cu/Zn superoxide dismutase homologs participate in Nicotiana benthamiana antiviral responses
Source: Front Microbiol. 2025 Jul 18;16:1561731. doi: 10.3389/fmicb.2025.1561731 (PMC12313585; doi:10.3389/fmicb.2025.1561731)
Supplement: Supplementary file 2 [file Table_1.docx]

**Table S1 Primers used in this study**

| **Name of primers** | **Sequence of primer（5’~3’）** | **Length of primer(nt)** | **Used for experiments** |
| --- | --- | --- | --- |
| NbCu/Zn-SOD -F | ATGGCCGCCCACACAATC | 18 | PCR  PCR |
| NbCu/Zn-SOD -R | TCATATTGGAGTCAAACCAAGTACTCCAC | 28 |  |
| BP/LR NbCu/Zn-SOD -F | GGGGACAAGTTTGTACAAAAAAGCAGGCTTCATGGCCGCCCACACAATC | 49 | PCR for Gateway |
| BP/LR NbCu/Zn-SOD -R | GGGGACCACTTTGTACAAGAAAGCTGGGTCTCATATTGGAGTCAAACCAAGTACTCCAC | 59 | PCR for Gateway |
| 1300GFP-NbCu/Zn-SOD-F | CAGGGTACCCGGGGATCCATGGCCGCCCACACAATC | 36 | Subcellular localization |
| 1300GFP-NbCu/Zn-SOD-R | CATGGTACTAGTGTCGACTATTGGAGTCAAACCAA | 35 | Subcellular localization |
| NbCu/Zn-SOD-1 qPCR-F | GCACGAGTTCGGTGACACTA | 20 | RT-qPCR |
| NbCu/Zn-SOD-1 qPCR-R | CTCAAGCTCGTGAACCACAA |  | RT-qPCR |
| NbCu/Zn-SOD-2 qPCR-F | CTTCACTACCACCAGCAGCA | 20 | RT-qPCR |
| NbCu/Zn-SOD-2 qPCR-R | GTGGCAGCAGAAAGTGTCAA | 20 | RT-qPCR |
| NbFe-SOD2-1 qPCR-F | GCAGGCTTGGAATCATCAAT | 20 | RT-qPCR |
| NbFe-SOD2-1 qPCR-R | ATGAGGATTTGACGCGTTTC | 20 | RT-qPCR |
| NbFe-SOD2-2 qPCR-F | AGAATGCTCTGGAGCCACAT | 20 | RT-qPCR |
| NbFe-SOD2-2 qPCR-R | TCCTCCTCCACTAGGCTTCA | 20 | RT-qPCR |
| NbFe-SOD2-3 qPCR-F | GGATGCTTTGGAGCCTCATA | 20 | RT-qPCR |
| NbFe-SOD2-3 qPCR-R | GGCTTCATTGATTCCCAGAA | 20 | RT-qPCR |
| NbMn-SOD-1 qPCR-F | GTGAGCAGACGGACCTTAGC | 20 | RT-qPCR |
| NbMn-SOD-1 qPCR-R | TCTCCTTTGGAAATGGCATC | 20 | RT-qPCR |
| NbFe-SOD2-4 qPCR-F | GGATGCTTTGGAGCCTCATA | 20 | RT-qPCR |
| NbFe-SOD2-4 qPCR-R | GGCTTCATTGATTCCCAGAA | 20 | RT-qPCR |
| NbCu/Zn-SOD-3 qPCR-F | GATTTCATGGCTTCCACGTC | 20 | RT-qPCR |
| NbCu/Zn-SOD-3 qPCR-R | CCATCTTCCCCAACTGTGAT | 20 | RT-qPCR |
| NbMn-SOD-2 qPCR-F | CGGCCATAGACACCAACTTT | 20 | RT-qPCR |
| NbMn-SOD-2 qPCR-R | TGCCAAAAAGAGGAACCAAG | 20 | RT-qPCR |
